# Alcohol dependence in a community sample of Aboriginal and Torres Strait Islander Australians: harms, getting help and awareness of local treatments

**DOI:** 10.1186/s13722-021-00274-2

**Published:** 2021-10-29

**Authors:** Teagan J. Weatherall, James H. Conigrave, Katherine M. Conigrave, Jimmy Perry, Scott Wilson, Robin Room, Tanya Chikritzhs, K. S. Kylie Lee

**Affiliations:** 1 Faculty of Medicine and Health, Discipline of Addiction Medicine, NHMRC Centre of Research Excellence in Indigenous Health and Alcohol, The University of Sydney, Sydney, NSW Australia; 2 The Edith Collins Centre (Translational Research in Alcohol Drugs and Toxicology), Sydney Local Health District, Sydney, NSW Australia; 3 Drug Health Services, Royal Prince Alfred Hospital, Camperdown, NSW Australia; 4 Aboriginal Drug and Alcohol Council South Australia, Underdale, SA Australia; 5 Centre for Alcohol Policy Research, La Trobe University, Bundoora, VIC Australia; 6 Department of Public Health Sciences, Centre for Social Research on Alcohol and Drugs, Stockholm University, Stockholm, Sweden; 7 National Drug Research Institute, Faculty of Health Sciences, Curtin University, Perth, WA Australia; 8 Burnet Institute, Melbourne, VIC Australia; 9 Faculty of Medicine and Health, Discipline of Addiction Medicine, Indigenous Health and Substance Use, NHMRC Centre of Research Excellence in Indigenous Health and Alcohol, The University of Sydney, King George V Building, 83-117 Missenden Road, Camperdown, NSW 2050 Australia

**Keywords:** Aboriginal and Torres Strait Islander, Indigenous, Alcohol dependence, Harms, Treatment

## Abstract

**Background:**

Few studies have examined links between current alcohol dependence and specific harms among Indigenous Australians. We investigated these associations as well as help seeking for drinking, awareness of local treatments and recommendations to help family or friends cut down or stop drinking in two Indigenous communities.

**Methods:**

A representative sample of Indigenous Australians was surveyed in one urban and one remote community in South Australia. Data were collected via the Grog Survey App. Participants were dependent if they reported two or more symptoms of alcohol dependence (ICD-11). Pearson chi-square tests were used to describe relationships between employment by gender, and dependence by awareness of medicines and local treatment options. Multivariate logistic regressions were used to predict the odds of dependent drinkers experiencing harms and getting help for drinking, controlling for age, gender, schooling and income.

**Results:**

A total of 775 Indigenous Australians took part in the study. After controlling for confounders, dependent drinkers were nearly eight times more likely to report a harm and nearly three times more likely to get help for their drinking—compared with non-dependent drinkers. Participants recommended accessing local support from an Aboriginal alcohol and other drugs worker, or a detoxification/ rehabilitation service.

**Discussion and conclusions:**

More support and funding is needed for Indigenous Australians to ensure local treatment options for dependent drinkers are readily available, appropriate and accessible. Involvement of local Aboriginal or Torres Strait Islander health professionals in delivery of care can help ensure that it is appropriate to an individual’s culture and context.

## Background

Globally, Indigenous Peoples who drink alcohol are at increased risk of developing dependence due to colonisation [1], government policies [2], ongoing trauma, and social disadvantage [3]. Globally, more Indigenous Peoples tend to be dependent (range: 3.8–16.6%; representative samples) compared to non-Indigenous people (2.6%) [4, 5]. Similarly, using established measurement criteria [6, 7], past studies with representative samples have indicated that slightly more Aboriginal and Torres Strait Islander (hereafter ‘Indigenous’) Australians are dependent compared to the general Australian population (2.2% versus 1.4%) [8, 9]. Indigenous Australians are aware of the impact alcohol has on them and their communities [10]. Impacts include a range of potential harms—physical, mental, social and emotional—to the drinker and to those around them [5]. To better reduce the harms from alcohol, it is important to improve our understanding of individuals who are dependent on alcohol, their reported harms, and access to treatment.

Many mainstream treatment options can benefit Indigenous Australians [11, 12]. However, culturally appropriate options are not always available, and this might make some Indigenous Australians feel uncomfortable, unwelcome, or prevent effective communication between health providers and Indigenous clients. The best way to provide primary care treatment for alcohol dependence for Indigenous Australians has had little study [13], and there is evidence to suggest that some Indigenous Australians might seek support outside routine healthcare settings. For example, people might seek help or advice for alcohol dependence from family or friends, Elders or spiritual leaders, the internet, or from culturally appropriate phone-based support [14].

Globally, Indigenous Peoples may experience greater barriers to getting help for alcohol dependence [15, 16]. These barriers include lack of information on local treatment options, stigma associated with alcohol dependence or with relapse prevention medicines [17], and treatments that may not be culturally-informed [18]. For the general Australian population, it can take 18 years for an individual to access help for alcohol use disorders [19]. While medicines to prevent relapse were first approved in Australia in 2003 [20, 21], they have had limited use [21]. For Indigenous Australians, there can be limited knowledge of or access to relapse prevention medicines or at-home withdrawal management [22, 23]. Additional challenges are a lack of services [24, 25] or treatment approaches that respect culture or are tailored for Indigenous contexts [22, 24].

Indigenous Australians who drink alcohol may have different patterns of drinking from the general population (e.g. episodic versus regular drinking, dependence or abstinence) [26, 27]. Accordingly, they may experience different harms and need access to different types of treatment. Globally, harms are more likely expected with dependence. However, Indigenous Australians who are heavy episodic drinkers (but not necessarily dependent), also report physical and social harms, and sometimes even more so than those who are dependent on alcohol (hereafter ‘dependent drinkers’) [10]. It cannot be assumed that Indigenous Australians who need help for their drinking are accessing help. This is likely due to historical traumas experienced by Indigenous Australians that may cause them to lose trust in the health care system [28]. Also, sometimes Indigenous Australians get help for drinking at the request of family, friends or legal reasons [29, 30]. It is important to understand who might benefit from treatment from primary health services (i.e. episodic heavy drinkers) versus those who might require treatment in specialised treatment services (i.e. dependent drinkers).

However, we are not aware of any population-level research that has looked at the harms experienced by Indigenous Australians who are dependent on alcohol compared to non-dependent drinkers. Additionally, it is not clear what support and treatment options Indigenous Australians are aware of or would recommend for alcohol dependence [13]. To address these gaps, we surveyed two community samples of Indigenous Australians to explore: (1) the magnitude of associations between alcohol dependence and harms; (2) whether dependent drinkers are more or less likely to get help for their drinking; and (3) whether the community are aware of local treatment options, and their recommendations on where family or friends can get help to cut down or stop drinking.

## Methods

### Aboriginal leadership

This study was designed by study investigators (including KL, KC, SW, RR, TC) in consultation with the Aboriginal Drug and Alcohol Council of South Australia (SW, JP). The lead author (TW) is an Australian Aboriginal woman from the Kamilaroi and Anaiwan nations with lived experience in both urban and remote Aboriginal communities.

### Ethical approval

Ethical approval was obtained from the Aboriginal Health Council of South Australia (Reference: 04/15/621) and as this was part of a larger study, from Metro South Health Human Research Ethics Committee in Queensland (Reference: HREC/16/QPAH/293).

### Setting

The study was conducted in two sites, one urban and one remote (community names are withheld to preserve their anonymity), in South Australia. There were no alcohol restrictions in the urban or remote sites. In the urban site, a sample of Indigenous Australians was drawn from an Indigenous area (as defined by the Australian Bureau of Statistics) [31], where more than 2% of residents were Indigenous Australian [31]. In the remote site, a sample of Indigenous Australians was drawn from a ‘very remote’ town as classified by the Australian Statistical Geography Standard [32], where more than 50% of residents were Indigenous Australian.

### Eligibility

Participants were Aboriginal and/or Torres Strait Islander, aged 16 years or older, and currently living in one of the two study sites (including people that had no fixed address).

### Recruitment

Research assistants confirmed each individual’s eligibility and set them up with an iPad and headphones. Participants completed the App anonymously. No names or birth dates were recorded. On completion, participants were reimbursed for their time with a store voucher ($20 for urban; $25 for remote—reflected higher cost of living). Research assistants received one day of face-to-face training in study methods and survey administration from study investigators (KL, KC). Recruitment has been described in detail elsewhere [8].

#### Urban

A quota-based convenience sample stratified by age, gender and socioeconomic status was used to recruit a representative sample [33]. Ten research assistants (7 Aboriginal, 3 non-Indigenous; 6 men and 4 women) collected survey data. Four Aboriginal research assistants resided and/or worked in the study site (JP) and another three were project officers (TW). Non-Indigenous staff included two project officers and a study investigator (KL). Recruitment took 36 days over 3.7 months (July, September to October 2019) and included a mix of planned events (in local services, community groups and public spaces) and unplanned events in public spaces (e.g. skate park, beach).

#### Remote

As there were only 57 eligible people [31] in the remote community, we aimed to recruit everyone. Four research assistants (3 Aboriginal, 1 non-Indigenous; 2 men, 2 women) collected survey data. The three Aboriginal research assistants were well known to the community and included two drug and alcohol workers and an Aboriginal health worker. The non-Indigenous staff member was a project officer. Recruitment took place on two occasions totalling 14 days (July/August, October 2019) and included a mix of planned events (e.g. barbeques at local women’s centre, council office, general stores) and unplanned events (e.g. driving around town).

### Data collection

#### Grog Survey App

Data were collected as part of a five-year Australian National Health and Medical Research Council project grant: The Grog Survey App (note: ‘grog’ is a commonly used term for alcohol). The overall aim of that larger project was to develop and test a tablet-based App to help Indigenous Australians describe their drinking (or abstinence). The App is an acceptable [34] and accurate [35] survey tool for Indigenous Australians and has been described in detail elsewhere [36].

The App collects information on demographics (including age, gender, language spoken, highest educational attainment, and individual income per week: < 200; 200–399; 400–599; 600–799; 800 + $AUD), alcohol consumption (modified Finnish method [37–39]), money spent on alcohol (0–25; 26–50; 51–75; 76–99; 100 + $AUD), frequency of symptoms of alcohol dependence (ICD-11), harms to self or others, treatment access, and participants’ feedback on using the App [36]. All survey data were collected offline and synchronised daily to a secure server hosted by the University of Sydney. The App can read out survey questions in plain English and Pitjantjatjara (an Aboriginal language commonly spoken in South Australia, Western Australia and the Northern Territory), in male and female audio.

#### Symptoms of current alcohol dependence

The term ‘current alcohol dependence’ is used in this study to refer to people who drink alcohol and are dependent (in the last 12-months). The World Health Organization’s International Classification of Diseases describes three key features of current alcohol dependence (ICD-11) [6]. To operationalise these for Indigenous Australian participants, Indigenous Australian community members were consulted, as were Indigenous (SW, JP) and non-Indigenous health professionals (KC) and researchers (KL, RR, TC).

Only participants who drank alcohol in the last 12 months (hereafter ‘current drinkers’) were asked dependence items. Participants were asked about: (1) Loss of control—“Some people feel like grog is the boss of them. In the last 12 months how often do you feel grog makes all the decisions (so you could not stop drinking, even if you tried)?”; (2) Alcohol withdrawal tremors (‘grog shakes’)—“Some people’s hands shake when they stop drinking or before their first drink of the day. In the last 12 months how often does this happen to you?”; and (3) Prioritising alcohol over other things—“Some people spend more time drinking than doing other things they need to do, like looking after family, culture or work. In the last 12 months how often does this happen with you?”. Responses for each item were indicated on a five-point Likert scale ranging from: (a) never; (b) ‘once in a blue moon’ (hardly ever, less than once a month); (c) sometimes (1–3 times a month); (d) weekly; to (e) most days or every day.

### Harms

Current drinkers were asked about harms to self or others from drinking: “Grog sometimes gives people worries or problems. In the last 12 months (that is from after (event name) last year until now) has grog given you any of these problems?”. Responses included: (a) Someone hit me; (b) I fell down; (c) I had a road accident; (d) My money runs out because it goes on grog; (e) The kids in my house get scared by my drinking; (f) I get into trouble with police or security guards. More than one harm could be selected, or the respondent could answer: (g) no.

#### Getting help

Current drinkers were asked: “Have you ever got help about drinking—like from a clinic, counsellor, rehab, detox or hospital?” (yes/no). If yes, current drinkers were then asked: “What made you get help?”. One response could be selected: (a) it was my choice; (b) family or friend pushed me; or (c) someone official pushed me (like welfare, police, or courts).

#### Awareness of or recommendations for local treatment options

All participants (including non-current drinkers and lifetime abstainers) were asked: “How far does a person need to travel to get to a rehab to stop drinking?” (‘rehab’ is commonly used to refer to an alcohol and other drug residential rehabilitation service). Responses were indicated using a slider (10 m to 3000 kms). The actual distance to the nearest Indigenous-specific residential rehabilitation service for the urban community was 300 km and 60 km for a mainstream service (one-way). For the remote site it was 800 km and 740 km, respectively for Indigenous-specific and mainstream services (one way).

All participants were asked two questions about relapse prevention medicines and grog shakes: “Can a person get a medicine to stop the grog shakes from a doctor around here?” and “If a person is finished with the grog shakes and wants to stay away from grog, can a doctor around here give them medicine to help?”. Responses included: (a) no; (b) yes; or (c) I don’t know.

All participants were asked: “If a friend or family wants to cut down or stop drinking, where should they get help?”. Responses included: (a) Aboriginal alcohol and drug worker; (b) Aboriginal health or mental health worker; (c) nurse or doctor; (d) counsellor; (e) friend, family; (f) Elder; or (g) minister or pastor (at least one response had to be selected, and more than one response could be selected). All participants were then asked: “Where else could they get help?”. Responses included: (a) day centre; (b) detox or rehab; (c) hospital; (d) AA or SMART Recovery groups; (e) men’s or women’s groups; (f) internet or phone helpline; or (g) other (at least one response had to be selected, and more than one response could be selected).

### Data analysis

Data cleaning and analysis were performed using R (version 4.0.4) [40]. Remote and urban samples were combined. A binary variable was constructed ('remoteness') to denote where participants were recruited from. This variable was used as a confounder in further analyses. Age was recoded into four categories (16–24, 25–44, 45–64, 65 + years old). Completion of highest level of educational attainment was recoded into six categories: university; TAFE (Technical and Further Education) or apprenticeship; Year 12; Year 11; Year 10; Year 9 or below. Participants were classified as likely dependent if they reported any two or more of the three key features of dependence, weekly or more frequently [6, 41]. This threshold was chosen based on face validity and likely impact of symptoms of this frequency on participants and their communities.

Demographic characteristics were described by drinking status (dependent drinkers, non-dependent drinkers, and non-current drinkers including lifetime abstainers), because alcohol consumption can vary greatly within and between communities [27]. We performed Pearson chi-square tests to describe sample characteristics, including, whether relationships exist between employment status and gender; awareness of medicines and location (responses: ‘no’ and ‘I don’t know’ were combined); awareness of medicines and dependence status; and awareness of or recommendations for local treatment options to cut down or stop drinking and dependence status.

Proportions of reported harms were described by dependence status (for current drinkers only, dependent compared with non-dependent drinkers). Logistic regressions were used to predict the odds of experiencing harms for dependent drinkers (1) compared to non-dependent drinkers (0); and to predict the odds of seeking help for drinking among dependent drinkers (1) compared to non-dependent drinkers (0). Logistic regressions controlled for age, gender, schooling, and individual income. Logistic regressions did not control for remoteness, due to their being only one remote dependent drinker.

## Results

### Participant characteristics

A total of 775 Indigenous Australians completed the App (urban: n = 706, 91.1%; remote: n = 69, 8.9%; Table 1). Approximately half of the participants were men (n = 365, 47.1%). Mean age was 38 years (SD = 16.1). More than three-quarters of participants had completed education beyond Year 10 (n = 607, 78.3%) and over a quarter were employed either full-time or part-time (n = 206, 26.6%). Demographics of dependent, non-dependent and non-current drinkers are presented in Table 1. One in 45 (n = 17, 2.2%) participants were likely dependent on alcohol and more likely to be men, have lower education, and be unemployed.

**Table 1:** Participant demographics by drinking status (n = 775)

Variable	Current drinkers^a^		Non-current
	Dependent^b^	Non-dependent	Drinkers^c^
	n = 17	n = 580	n = 178
	n (%)	n (%)	n (%)
Remoteness			
Urban	16 (94.1%)	538 (92.8%)	152 (85.4%)
Remote	1 (5.9%)	42 (7.2%)	26 (14.6%)
Age groups			
16–24	2 (11.8)	158 (27.2)	40 (22.5)
25–44	7 (41.2)	255 (44.0)	42 (23.6)
45–64	8 (47.1)	140 (24.1)	68 (38.2)
65 +	0 (0.0)	27 (4.7)	28 (15.7)
Gender			
Female	6 (35.3)	294 (50.7)	110 (61.8)
Male	11 (64.7)	286 (49.3)	68 (38.2)
Level of highest educational attainment			
University	0 (0.0)	30 (5.2)	8 (4.5)
TAFEd or apprenticeship	1 (5.9)	133 (22.9)	43 (24.2)
Year 12	1 (5.9)	87 (15.0)	15 (8.4)
Year 11	3 (17.6)	105 (18.1)	29 (16.3)
Year 10	4 (23.5)	115 (19.8)	33 (18.5)
Year 9 or below	8 (47.1)	110 (19.0)	50 (28.1)
Employment status			
Full-time	0 (0.0)	142 (24.5)	22 (12.4)
Part-time	1 (5.9)	36 (6.2)	5 (2.8)
Casual	0 (0.0)	29 (5.0)	2 (1.1)
Work for the Dole/CDP^e^	1 (5.9)	7 (1.2)	2 (1.1)
Other	0 (0.0)	4 (0.7)	1 (0.6)
Not employed	15 (88.2)	362 (62.4)	146 (82.0)
Individual weekly income ($AUD)			
< 200	4 (23.5)	108 (18.6)	35 (19.7)
200–399	4 (23.5)	151 (26.0)	53 (29.8)
400–599	6 (35.3)	128 (22.1)	44 (24.7)
600–799	1 (5.9)	68 (11.7)	20 (11.2)
> 800	2 (11.8)	125 (21.6)	26 (14.6)

^a^Individuals who had any alcohol in the last 12-months

^b^Reported two or more symptoms of alcohol dependence, weekly or more frequently (ICD-11)

^c^Individuals who did not have any alcohol in the last 12-months, including lifetime abstainers

^d^TAFE: Technical and Further Education

^e^CDP: Community Development Program (Australian Government remote employment and development service)

### Association between harms and current alcohol dependence (n = 597)

More than a quarter of all current drinkers reported at least one harm from alcohol in the last 12-months (n = 162, 27.1%; Table 2). The most reported harm was spending too much money on alcohol (n = 68, 11.4%). Of the dependent drinkers, three-quarters reported at least one harm in the last 12-months (n = 13/17, 76.5%), with the most reported harm being getting into trouble with police or security guards (n = 7, 41.2%). No dependent drinkers reported falling down. One in four non-dependent drinkers reported at least one harm in the last 12-months (n = 149/580, 25.7%), with the most reported harm being spending too much money on grog (n = 64, 11%).

**Table 2:** Frequency of self-reported harms among dependent and non-dependent drinkers^a^ (n = 597)

	Dependent^b^ n = 17	Non-dependent n = 580	Total n = 597
	n (%)	n (%)	n (%)
Reported a harm	13 (76.5)	149 (25.7)	162 (27.1)
Specific harms^c^			
Someone hit me	2 (11.8)	40 (6.9)	42 (7.0)
Fell down	0	45 (7.8)	45 (7.5)
Had road accident	1 (5.9)	6 (1.0)	7 (1.2)
Money goes on grog	4 (23.5)	64 (11.0)	68 (11.4)
Kids scared	5 (29.4)	17 (2.9)	22 (6.7)
Trouble with police	7 (41.2)	33 (5.7)	40 (3.7)

^a^Individuals who had any alcohol in the last 12-months

^b^Reported two or more symptoms of alcohol dependence, weekly or more frequently (ICD-11)

^c^More than one specific harm could be selected

In a series of multivariable logistic regressions, we examined links between alcohol dependence and reported harms adjusting for demographics (age, gender, schooling, and individual income). Adjusted and unadjusted estimates were similar. Due to there being few dependent drinkers in our sample (n = 17/597), there was a large degree of uncertainty in our findings i.e., confidence intervals were wide. As shown in Table 3, after adjustment, dependent drinkers were nearly eight times more likely than non-dependent drinkers to report a harm (OR = 7.84; 95% CI 2.64, 28.73). Dependent drinkers were ten times more likely to report children in their house being scared by their drinking (OR = 10.29; 95% CI 2.82, 33.33) and 11 times more likely to report trouble with police or security guards (OR = 11.17; 95% CI 3.67, 32.68). Three harms were not significantly associated with dependence (‘someone hit me’, ‘had a road accident’ and ‘money goes on grog’).

**Table 3:** Odds of self-reported alcohol-related harms among alcohol dependent drinkers compared to non-dependent drinkers (n = 597)

Outcome	OR [95% CI] (unadjusted)	OR [95% CI] (adjusted)
Reported any harm	9.4 [3.27, 33.78]*	7.84 [2.64, 28.73]*
Specific harms		
Someone hit me	1.8 [0.28, 6.68]	1.6 [0.24, 6.15]
Had road accident	5.98 [0.31, 37.94]	5.24 [0.25, 40.3]
Money goes on grog	2.48 [0.68, 7.25]	1.98 [0.51, 6.15]
Kids scared	13.8 [4.03, 42.04]*	10.29 [2.82, 33.33]*
Trouble with police	11.6 [3.99, 32.22]*	11.17 [3.67, 32.68]*

*p < 0.05; All coefficients describe the effect of being a dependent drinker (1) compared to a non-dependent drinker (0); Odds ratios were calculated using logistic regressions; OR (controlled) = logistic regression results controlled for age, gender, schooling and individual income; Age = age in years; School = years of completed schooling (from completed no schooling to Year 12); Individual income = weekly income categories ($AUD: < 200; 200–399; 400–599; 600–799; 800 + ; recoded 1–5); Only current drinkers were included in analysis; Alcohol dependence = reported two or more symptoms of alcohol dependence (ICD-11; in the last 12-months), weekly or more frequently; There was insufficient data to estimate the value of the harm, “I fell down” (so this variable was omitted from this table)

### Association between getting help and current alcohol dependence (n = 597)

More than one in five current drinkers reported getting help for their drinking at some time in their life (n = 133/597, 22.3%; non-dependent: n = 124/580, 21.4%; dependent: n = 9/17, 52.9%). Of those who received help for their drinking, the majority were men (n = 83/297, 62.4%). Among dependent drinkers, reasons for getting help were evenly split across: ‘it was my choice’, ‘family/ friends pushed me’ or ‘someone official pushed me’ (each n = 3). Among non-dependent drinkers, ‘it was my choice’ was the most commonly reported reason for getting help (n = 86/124, 69.4%).

As shown in Table 4, after controlling for confounders, dependent drinkers were nearly three times more likely than non-dependent drinkers to get help for their drinking (OR = 2.96; 95% CI 1.06, 8.38). The patterns of results were similar for unadjusted and adjusted odds ratios (controlling for age, gender, schooling, and individual income). Due to the large uncertainty resulting from the small number of dependent participants it was not clear if dependent people sought help for different reasons than non-dependent people.

**Table 4:** Odds of seeking help for drinking among alcohol dependent drinkers compared to non-dependent drinkers (n = 597)

Outcome	OR [95% CI] (uncontrolled)	OR [95% CI] (controlled)
Got help for drinking	4.14 [1.55, 11.23]*	2.96 [1.06, 8.38]*
Reasons for help seeking help (n = 133)		
Why: “It was my choice”	0.22 [0.04, 0.88]*	0.24 [0.05, 1.01]
Why: “Family/friends pushed me”	1.8 [0.36, 7.29]	1.79 [0.34, 7.89]
Why: “Someone official pushed me”	5.14 [0.98, 22.58]*	5.39 [0.77, 33.71]

*p < 0.05; All coefficients describe the effect of being a dependent drinker (1) compared to a non-dependent drinker (0); Odds ratios were calculated using logistic regressions; OR (controlled) = logistic regression results controlled for age, gender, schooling and individual income; Age = age in years; School = years of completed schooling (from completed no schooling to Year 12); Individual income = weekly income categories ($AUD: < 200; 200–399; 400–599; 600–799; 800 + ; recoded 1–5); Only current drinkers were included in analysis; Alcohol dependence = reported two or more symptoms of alcohol dependence, weekly or more frequently (ICD-11; in the last 12-months); Got help about drinking = “Have you ever got help about drinking—like from a clinic, counsellor, rehab, detox or hospital?”; 133 current drinkers said yes, they got help for their drinking

### Awareness of or recommendations for local treatment options to help Indigenous Australians cut down or stop drinking (n = 775)

#### Residential rehabilitation services (n = 775)

Awareness varied widely as to where the nearest residential rehabilitation services were, either Indigenous-specific or mainstream. Responses ranged from 0–3000 km.

Of the 706 urban participants, just 24 (3.5%; all of whom were non-dependent or non-current drinkers) correctly estimated that a residential rehabilitation service was 50–75 km from the urban site (a mainstream service was 60 km away). Just six individuals (0.8%; all of whom were non-dependent or non-current drinkers) estimated that a residential rehabilitation service was 200–400 km away (an Indigenous-specific service was 300 km away). Most dependent drinkers (n = 13/16, 81.2%) overestimated the availability of residential rehabilitation services, reporting that a residential rehabilitation service was within 25 km of their community.

Of the 69 remote participants, just ten (14.5%; all of whom were non-dependent or non-current drinkers) estimated that a residential rehabilitation service was 650–900 km from the remote site (a mainstream service was 740 km away and an Indigenous-specific service was 800 km away). The one dependent drinker in this site said that a residential rehabilitation service was 5 km from the remote site.

#### Medicines to stop grog shakes or stay away from alcohol

**Fig. 1:**
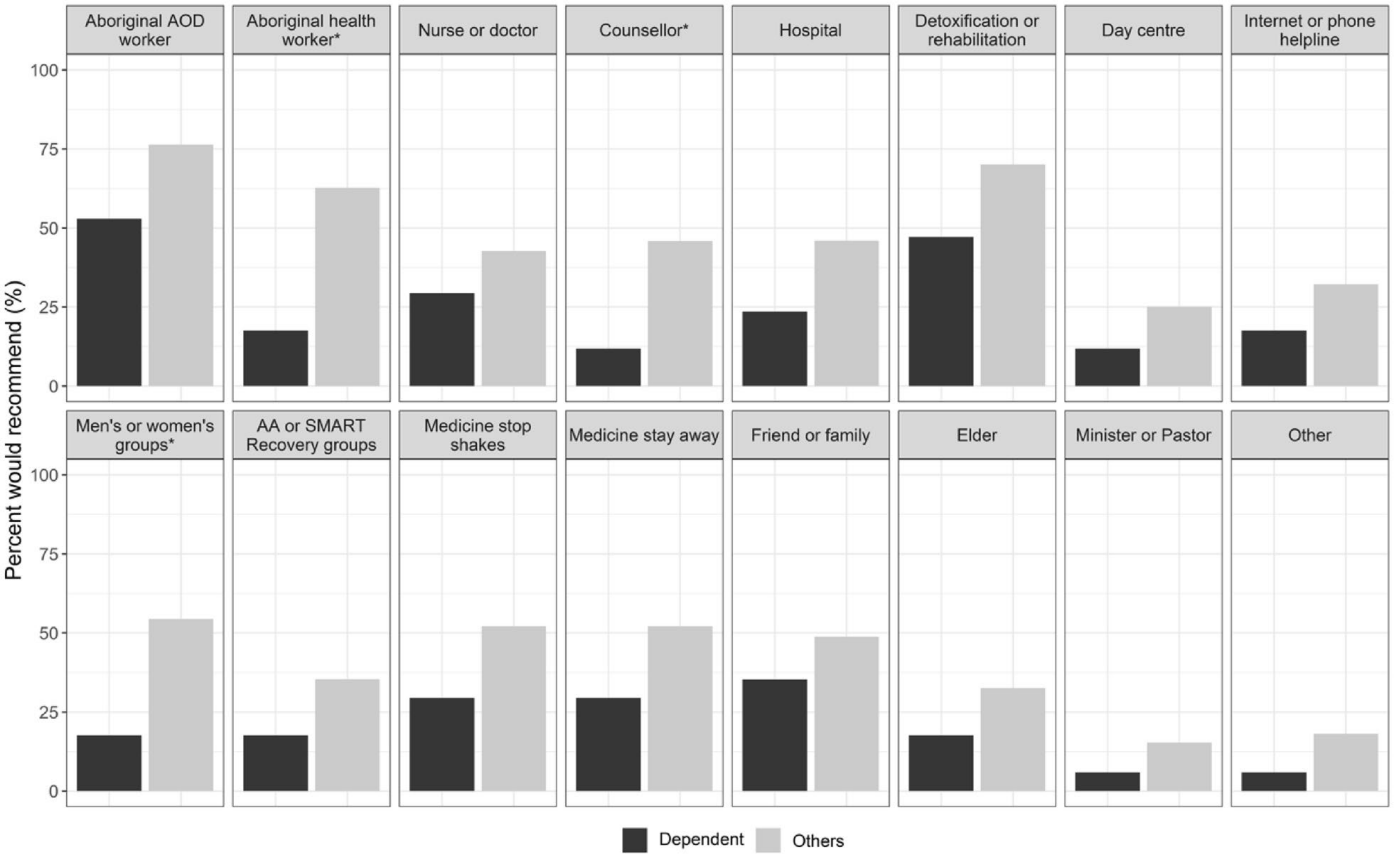
Where Indigenous Australians recommend others can access local support, stratified by drinking status (n = 775). *p < 0.05, statistically significant based on chi-square tests; Alcohol dependence = reported two or more symptoms of alcohol dependence, weekly or more frequently (ICD-11; in the last 12-months); Others = non-dependent drinkers, individuals who have not had any alcohol in the last 12-months and lifetime abstainers; Aboriginal AOD worker = Aboriginal alcohol and other drugs worker; AA = Alcoholics Anonymous (mutual support programme); SMART Recovery = Self Management and Recovery Training (mutual support programme); Medicine to stop shakes = diazepam; Medicine to stay away = relapse prevention pharmacotherapies (i.e. naltrexone or acamprosate)

More than a quarter of dependent drinkers (n = 5/17, 29.4%) and half of the non-dependent or non-current drinkers (n = 395/758, 52.1%), said that medicines to stop ‘grog shakes’ could be accessed from a local doctor (Fig. 1). But this difference was not statistically significant (X^2^ (1, n = 775) = 2.6, p = 0.11). Similarly, perceived accessibility of medicines to stop grog shakes did not vary by remoteness (X^2^ (1, n = 775) = 0.6, p = 0.43).

Similar proportions of dependent drinkers and non-dependent or non-current drinkers said that medicines ‘to stay away from grog’ could be accessed from a local doctor (n = 7/17, 41.2%; n = 328/758, 43.3%). Perceived accessibility of these relapse prevention medicines did not vary by dependence status (X^2^ (1, n = 775) = 0, p = 1) or by remoteness (X^2^ (1, n = 775) = 0.7, p = 0.4).

#### Other supports to help friends or family cut down or stop drinking

The two most recommended supports to help friends or family cut down or stop drinking were: an Aboriginal alcohol and other drugs worker, and detoxification/ rehabilitation service (Fig. 1). Non-dependent and non-current drinkers were more likely than dependent drinkers to recommend accessing support for drinking from an Aboriginal health worker (X^2^ (1, n = 775) = 12.42, p =  < 0.001), counsellor (X^2^ (1, n = 775) = 6.51, p = 0.011), or men's or women's groups (X^2^ (1, n = 775) = 7.60, p = 0.006; Fig. 1).

## Discussion

We aimed to explore if Indigenous Australians who met the criteria for current alcohol dependence are more or less likely to experience harms and access support. We found that dependent drinkers were more likely to report harms from drinking and were also more likely to have had treatment for their drinking. Participants were most likely to recommend getting help from an Aboriginal alcohol and drug worker, or a detoxification/residential rehabilitation service. However, among the entire community sample, dependent drinkers were less likely to select any recommendations for help for drinking compared to non-dependent current drinkers and non-drinkers. It is important that early intervention and treatments are available, appropriate, and accessible for Indigenous Australians.

### Association between harms and current alcohol dependence

We found dependent drinkers were nearly eight times more likely than non-dependent drinkers to report an alcohol-related harm. Dependent drinkers were more likely to report children in their house being scared by their drinking and more likely to report having trouble with police or security guards. It is well known that harms from drinking go beyond the drinker, they affect children, families and communities [42]. In Indigenous Australian contexts, social issues may also contribute to family harms experienced [43]. For example, overcrowding in houses [1] may mean there are more people who drink alcohol in a house and more shared alcohol available—all with children in the home. Overcrowded houses also potentially encourage dependent drinkers to consume alcohol in public places, such as parks and beaches. As dependent drinkers typically consume more alcohol and more often, this can lead to antisocial behaviours involving security or police [44]. Behaviours such as public drunkenness are common in many cities in general in Australia. However, Indigenous Australians may be targeted by police because of social disadvantage, and at times because of discrimination and racism [45]. More support is needed to help individuals, families and whole communities to address past traumas and social issues that are linked to or exacerbated by alcohol-related harms [1, 46, 47].

### Association between getting help and current alcohol dependence

Even though drinking can harm their loved ones, barriers may prevent some Indigenous Australians from getting help for their drinking. Also, some people may not feel comfortable accessing help for drinking at mainstream services. For example, many Indigenous Australians experience trauma from past government policies (e.g. the forced removal of children) [10]. Consequently, individuals may be concerned that their children will be taken away if they disclose any risky drinking [48]. Despite this, when compared to non-dependent drinkers, dependent drinkers were approximately three times more likely to receive help for their drinking. It is positive that in this community, Indigenous Australians who were dependent on alcohol sought help despite many barriers to accessing treatment and past experience of traumas. In the general Australian population, it can take up to 14 years for an individual to seek treatment for alcohol dependence [19]. In Indigenous Australian contexts, reasons for seeking help vary from because they want to, at the request of others (e.g. family, friends, government officials) [49], or because of pressure from others to address their drinking [50, 51]. Individuals may not always recognise the need to get help for their drinking [52]. Communities would benefit from improved screening, brief interventions and health promotion initiatives offered by local Aboriginal Community Controlled Health Organisations and suitable mainstream services [10, 53]. To achieve this, specific alcohol-related government funding and support would be needed in every Indigenous community, given the varying patterns of drinking within and between communities [22, 54, 55].

### Awareness of or recommendations for local treatment options

Participants were more likely to recommend getting help for alcohol from either an Aboriginal alcohol and drug worker, or detoxification/ residential rehabilitation service. While drug and alcohol counsellors were highly recommended by Indigenous Australians in our sample, they may be difficult to access due to long waiting-lists [56]. The majority of participants did not know how far away a residential rehabilitation service was from their community. Some treatment options (e.g. residential rehabilitation services) may be hundreds of kilometres away from some communities [29]. Also, there could be a gap between what treatment dependent drinkers need or want and what is actually available locally [24].

Most participants believed that relapse prevention medicines could be accessed from a local doctor. However, while participants in our sample knew that medicines to stop tremors or prevent relapse were available, they may not understand how they work, nor feel confident to take them [57]. We identified that dependent drinkers were less likely to recommend any kind of help than others. This could reflect negative experiences in the health system, a lack of a desire for help, doubts about being able to make changes, or not understanding available supports [22]. Indigenous communities across Australia need treatment options that are readily available, readily accessible and culturally appropriate [24, 58].

### Implications for policy, practice and research

Alcohol-related policies aimed at Indigenous Australians need meaningful and active engagement from local Indigenous community members, leaders and elders [59]. Local treatment options should be tailored to reflect Indigenous values—strong families, caring for Country and belonging [43]. More work is needed to support health services to improve alcohol screening and provide alcohol brief interventions to Indigenous clients [60]. Community-level surveys could be used to monitor the prevalence of alcohol dependence and potential treatment needs in larger representative samples of Indigenous Australians [61]. Future research is needed to identify the effectiveness of brief interventions, treatment in different settings, and other supports—all guided by local community members, Elders and local cultural protocols.

### Limitations

This study was based on self-reported data from two Indigenous communities located in one Australian state and may not generalisable to other communities. The urban and remote samples were combined in some analyses, however, there were little differences between these two communities with regards to schooling, licensed venues, and alcohol restrictions. The remote community had only one dependent drinker, so analyses were not adjusted for remoteness. The number of dependent drinkers was small (n = 17), resulting in wide confidence intervals. Larger scale studies of representative samples of Indigenous Australians would be useful for more accurate estimates of the associations between dependence and harms and treatment access.

## Conclusion

Using a representative sample of Indigenous Australians from two communities, we found that dependent drinkers were more likely to report harms and more likely to have received help for their drinking. However, dependent drinkers were less likely to recommend available support than others. More support is needed to help communities address past traumas and social issues that may be linked to alcohol-related harms [46] and barriers to getting help for drinking. Communities also need increased awareness of available local treatment options for alcohol dependence. Finally, investment in treatment and support for alcohol dependence by governments and developed with Indigenous community members [59] could help ensure that local treatment options are available, accessible and appropriate.

## Data Availability

Data for this project is stored at the University of Sydney based at Drug Health Services, KGV Building, Missenden Road, Camperdown, New South Wales, 2050, Australia.

## Abbreviations

AA: Alcoholics Anonymous (mutual support programme)
ACHSA: Aboriginal Health Council of South Australia
AOD: Alcohol and other drugs
CDP: Community Development Program (Australian Government remote employment and development service)
CI: Confidence intervals
DF: Degrees of freedom
DSM: American Psychiatric Association’s Diagnostic and Statistical Manual
ICD: World Health Organization’s International Classification of Diseases
JC: James H. Conigrave
JP: Jimmy Perry
KC: Katherine M. Conigrave
KL: K.S. Kylie Lee
OR: Odds ratio
RR: Robin Room
SD: Standard deviation
SMART Recovery: Self Management and Recovery Training (mutual support programme)
SW: Scott Wilson
TAFE: Technical and Further Education
TC: Tanya Chikritzhs
TW: Teagan J. Weatherall
